# Determination of Paracetamol on Electrochemically Reduced Graphene Oxide–Antimony Nanocomposite Modified Pencil Graphite Electrode Using Adsorptive Stripping Differential Pulse Voltammetry

**DOI:** 10.3390/s22155784

**Published:** 2022-08-03

**Authors:** Zandile D. Leve, Nazeem Jahed, Nelia A. Sanga, Emmanuel I. Iwuoha, Keagan Pokpas

**Affiliations:** SensorLab, Chemistry Department, University of the Western Cape, Cape Town 7535, South Africa; 3135207@myuwc.ac.za (Z.D.L.); njahed@uwc.ac.za (N.J.); 3471286@myuwc.ac.za (N.A.S.); eiwuoha@uwc.ac.za (E.I.I.)

**Keywords:** electrochemically reduced graphene oxide–antimony nanocomposite, graphene oxide, pencil graphite electrode, paracetamol, adsorptive stripping differential pulse voltammetry

## Abstract

A simple, highly sensitive, accurate, and low-cost electrochemical sensor was developed for the determination of over-the-counter painkiller, paracetamol (PC). The enhanced sensing capabilities of the developed sensor were fabricated by the single-step modification of disposable pencil graphite electrodes (PGEs) with the simultaneous electrochemical reduction in graphene oxide and antimony (II) salts. For this purpose, an electrochemically reduced graphene oxide–antimony nanoparticle (ERGO-SbNP) nanocomposite material was prepared by trapping metallic nanoparticles between individual graphene sheets in the modification of PGEs. Structural characterization by FTIR and Raman spectroscopy was employed to confirm the presence of oxygen functional groups and defects in the conjugated carbon-based structure of GO. Morphological differences between the modified PGEs were confirmed by HRTEM and HRSEM for the presence of nanoparticles. The modified electrodes were further electrochemically characterized using CV and EIS. The electrooxidation of PC on an ERGO-SbNPs-PGE was achieved by adsorptive stripping differential pulse voltametric analysis in 0.1 mol·L^−1^ phosphate buffer solution at pH = 7.0. The optimum current response was used to record a detection limit of 0.057 µmol·L^−1^ for PC. The electrochemical sensor was further used in real sample analysis for a commercially available pharmaceutical tablet (500 mg PC), for which the percentage recovery was between 99.4% and 100.8%.

## 1. Introduction

Paracetamol (*N*-acetyl-*p*-aminophenol) is a synthetic non-opioid analgesic and antipyretic drug. In general, its analgesic and antipyretic capabilities are equivalent to those of non-steroidal antiinflammatory drugs (NSAIDs) [1]. The main reactive metabolite of its oxidation is the *N*-acetyl-*p*-benzoquinone imine (NAPQI). *N*-acetyl-*p*-benzoquinone imine is hepatoxic but is normally rapidly converted to an inert cysteine and/or mercapturic acid metabolite by conjugation with glutathione [2]. However, build-up of the toxic metabolite due to overdose may take place, which can cause liver and kidney failure [1], as well as skin rashes and pancreas inflammation [3]. Therefore, there is an opportunity for the development of novel sensors that are simple, highly sensitive, stable, and accurate, and can detect at the lowest analytical limits. Various methods developed for the determination of paracetamol have been reported and these include capillary electrophoresis (CE) [4], high-performance liquid chromatography (HPLC) [5], UV-spectrophotometry [6], HPLC-tandem mass spectrometry [7], and thermogravimetric analysis (TGA) [8]. The determination of paracetamol using these methods exhibits high sensitivities, but they are generally performed at centralized laboratories and involve tedious procedures. These may limit their application for immediate determination [3]. 

Electrochemical methods have been strongly considered for the determination of paracetamol as an alternative as they are considered selective, highly sensitive, inexpensive, less time-consuming, and of a wide dynamic range and quick response [9]. These attributes make electrochemical methods more common for the study of electroactive compounds in pharmaceutical forms and physiological fluids. The determination of paracetamol by graphene-based and electrochemical sensors has been well-documented in the previous literature. Görҫay et al. [9] prepared a novel derivative formazan modified pencil graphite electrode (PGE) for the determination of paracetamol. Bis(3-(2-hydroxyphenyl)-1-phenylformazan (BF) was synthesized and electrodeposited onto a PGE in which the sensor was used to detect paracetamol where the LOD was found to be 2.4 nmol·L^−1^ (S/N = 3) [9]. An electrochemical sensor based on a reduced graphene oxide–silver nanocomposite was designed by Tavakolian and Tashkhourian and was used to detect paracetamol. The rGO-Ag nanocomposite was modified onto a carbon paste electrode (CPE) and the LOD was 1.8 µmol·L^−1^ for paracetamol under optimized conditions [10]. Similarly, an Fe_3_O_4_/rGO composite fabricated onto a glassy carbon electrode (GCE) by Thi Anh Thu et al. obtained an LOD of 0.72 µmol·L^−1^ for paracetamol [11].

Paracetamol is an electroactive, phenolic compound that presents sluggish voltammetric responses on traditional electrodes [12,13]. Phenolic compounds can foul solid-state electrodes as their oxidation results in tarry deposits being formed on the electrode surface, thus resulting in irreversible polymerization products [14,15,16]. Graphene greatly promotes the electrochemical reactivity of biomolecules on modified surfaces due to its extraordinary electronic transport properties and high electrocatalytic activity. Furthermore, the unique two-dimensional crystal structure of graphene makes it extremely attractive as a support material for metal and metal oxide catalyst nanoparticles [17]. Antimony-based electrodes have also attracted attention in the development of electrochemical sensors due to their intrinsic properties, such as a negative over-potential to hydrogen evolution and large scan potential range, and because they can be used in acid solutions and present a low stripping current signal for themselves [18]. Miller [19] reported that antimony is a semimetal whose band structure is characteristic of that of bismuth, which also shares similarities in properties and can serve as “mercury-free” electrode material in heavy metals’ stripping analysis [20]. In addition, the energy overlap between the conduction and valence bands is about 180 meV at 4.2 K [19].

The modification of graphene with SbNPs has been shown to provide great prospects for new functions in sensing, energy, and other applications due to the advantageous synergistic effects of the component materials, thereby expanding the application area of graphene [21]. The synergistic effect of graphene with platinum group, noble, and other nanomaterials has been widely explored in the literature in diverse contexts [22,23]. Piovesan et al. proposed a modified glassy carbon electrode based on an rGO and AuNPs nanocomposite for the determination of endocrine disruptor, methylparaben [22]. A screen printed electrode surface was modified with an NiCo_2_O_4_/rGO nanocomposite, which was used to detect hydroylamine [23], among others. Electrodes based on reduced graphene oxide modified with SbNPs have previously been studied for the detection of organic compounds [21,24]. Cesarino et al. [21] successfully applied rGO-SbNPs dispersed in water and drop-casted onto GCE to determine estriol hormone in water, which yielded an LOD of 0.5 nmol·L^−1^ [21]. Furthermore, Da Silva et al. [24] evaluated SbNPs/rGO and CuNPs/rGO composites on GCE surfaces for the oxidation of levofloxacin using DPV, and the LODs were found to be 4.1 × 10^−8^ mol·L^−1^ and 1.7 × 10^−8^ mol·L^−1^, respectively [24]. Our research group previously fabricated a sensor based on electrochemically reduced graphene oxide modified on an in situ plated bismuth pencil graphite electrode [25] and, recently, an in situ plated thin mercury film modification [26] was achieved for the detection of trace metals in water. In this paper, a GO-Sb dispersion was electrochemically deposited onto a pencil graphite electrode following an electrochemical reduction step to develop an electrochemically reduced graphene oxide–antimony nanocomposite modified pencil graphite electrode (ERGO-SbNPs-PGE). Antimony nanoparticles trapped within the reduced graphene oxide sheets create a nanocomposite material combining the properties of the metallic nanoparticle with the 2D carbon nanostructure, and this was confirmed by microscopic and spectroscopic analysis. The synthesized ERGO-SbNPs-PGE sensor, after characterizations and optimizations, was used for the quantitative determination of paracetamol via adsorptive stripping differential pulse voltammetric analysis. The optimized sensor was used for the quantification of paracetamol in a commercially available pharmaceutical tablet. 

## 2. Materials and Methods

### 2.1. Reagents and Chemicals

All chemicals used for experiments were of analytical grade and stock solutions were prepared using ultrapure water where needed. Graphite powder (<150 µm, 99.99% trace metals basis), sulphuric acid (95–97%, Sigma-Aldrich, Darmstadt, Germany), and potassium permanganate were obtained from PAL Machinery and Chemicals, along with hydrogen peroxide (Sigma-Aldrich). Antimony stock solution (1.000 mg. L^−1^, atomic absorption standard solution) was obtained from Sigma-Aldrich. Acetate buffer solution (0.1 mol·L^−1^, pH 4.6) was prepared by mixing acetic acid and sodium acetate (Sigma-Aldrich), and phosphate buffer solution (0.1 mol·L^−1^, pH 7.02) was prepared from diluting sodium phosphate monobasic and sodium phosphate dibasic solutions (1 mol·L^−1^), with both buffers diluted in ultrapure water. A paracetamol solution was prepared by dissolving the powder in 0.1 mol·L^−1^ phosphate buffer solution (pH = 7.02). Hydrochloric acid (Sigma-Aldrich) was diluted to make up 1 mol·L^−1^ and nitric acid (Sigma-Aldrich) to 3 mol·L^−1^ solutions. Solutions were diluted as required with ultrapure water from the Millipore system.

### 2.2. Instrumentation

Fourier transform infrared spectroscopy data were collected from a Nexus 670 FTIR spectrometer. The Raman spectroscopy information was captured using a Dilor XY Raman spectrometer with a Coherent Innova 300 Argon laser composed of a 514.9 nm laser excitation. The samples for HRTEM were analyzed using a Tecnai G2 F20X-Twin MAT Field Emission Transmission Electron Microscope from FEI (Eindhoven, Netherlands) under an acceleration voltage of 200 kV. The HRSEM images were obtained using a LEO 1450 SEM 30 kV instrument equipped with Electronic Data System (EDS) and Windows Deployment Services (WDS). All voltammetric measurements (cyclic and differential pulse voltammetry) were conducted on a 797 VA Computrace (Metrohm, Switzerland) that was connected to a personal computer. The instrument was composed of a three-electrode electrochemical system that included Ag/AgCl with 3 mol·L^−1^ KCl solution as a reference electrode, platinum wire as an auxiliary electrode, and the bare PGE, ERGO-PGE, and ERGO-SbNPs-PGE as a working electrode. Pencil graphite electrode (Pentel, HB with 0.5 mm diameter and 60 mm length) was inserted into a fabricated mechanical holder. Only 10 mm length (geometric surface area of 16.09 mm^2^~1.609 cm^2^) was used for measurements in supporting electrolyte. 

### 2.3. Synthesis of GO

Graphene oxide was synthesized according to modified Hummers’ method [27]. The experiment proceeded by addition of 2 g graphite into 50 mL H_2_SO_4_ in a conical flask stirred under ice bath. Addition of 7 g KMnO_4_ followed, and after completion the flask transferred into a warm bath at 37 °C for 2 h. Then 100 mL of ultrapure water was added followed by 5 mL 30% H_2_O_2_ under ice bath. Thereafter the contents of the conical flask were filtered through the Büchner funnel and successively washed with 10% HCl and ultrapure water. The resulting graphite oxide powder was placed under vacuum drying for 48 h. Graphene oxide solution was prepared by dispersing graphite oxide in 0.1 mol·L^−1^ acetate buffer solution to make up 1.0 mg.mL^−1^ dispersion and ultrasonicated for 1.5 h.

### 2.4. Preparation of ERGO-PGE and ERGO-SbNPs-PGE

Prior to modification electrodes were electrochemically treated. Pencil graphite electrodes were cleaned with 3 mol·L^−1^ HNO_3_ and washed thoroughly with ultrapure water. Electrochemical treatment proceeded in 0.1 mol·L^−1^ acetate buffer solution using CV at a scan rate of 50 mV·s^−1^. A 150 µL aliquot of antimony standard solution (1000 µg.mL^−1^, atomic absorption standard) was dispersed in 1.0 mg·mL^−1^ GO dispersion and ultrasonicated for 1 h. The final dispersion along with that of GO were electrodeposited onto electrochemically treated PGEs using CV in the potential range between −1.5 and +0.3 V for five successive cycles. Electrodeposition procedure proceeded with following parameters; deposition potential (−0.7 V), deposition time (120 s), voltage step (0.005 V), and scan rate (100 mV·s^−1^). The resulting ERGO-PGE and ERGO-SbNPs-PGE were dried in the refrigerator (−4 °C) overnight.

### 2.5. Adsorptive Stripping Differential Pulse Voltammetric Analysis

Commercial drug containing 500 mg PC was used to evaluate analytical performance of ERGO-SbNPs-PGE. The PC tablet was dissolved in 0.1 mol·L^−1^ phosphate buffer solution (pH 7.0). Adsorptive stripping differential pulse voltammetry was used for the analytical studies. The PC in solution was stirred at 1000 rpm for 60 s, which allows for the preconcentration of analyte (PC) close to the electrode. Differential pulse voltammetry parameters were as follows; pulse amplitude = 0.05005 V, voltage step time = 0.4 s, scan rate = 0.0126 V·s^−1^ (12.6 mV·s^−1^).

## 3. Results and Discussion

### 3.1. Structural and Morphological Characterizations of ERGO-SbNPs

**Figure 1:**
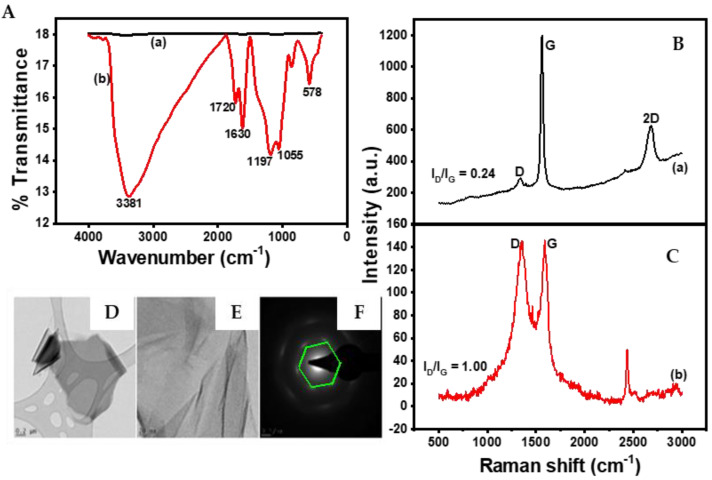
Structural and morphological characterizations of graphite (a) and GO (b); FTIR (**A**), Raman spectra of graphite (**B**) and GO (**C**), and HRTEM of graphite (**D**) and GO (**E**) with accompanying SAED of GO and highlighted diffraction pattern (**F**).

The chemical and structural compositions of graphene oxide synthesized from graphite were studied through Fourier transform infrared (FTIR) spectra analysis. The characteristic absorption bands of graphite and GO were exhibited by FTIR spectra as shown in Figure 1A. The graphite (curve a) FTIR spectrum in Figure 1A shows the absorbance of significant peaks, which is attributed to the chemical inertness of bulk graphite [28]. However, the FTIR spectrum of GO (curve b) shows variation, with a strong distinct band found at 3381 cm^−1^, which is due to the O-H stretching vibration validating the presence of the OH group in the structure. It may also indicate the presence of the COOH group within the structure. The peak at 1720 cm^−1^ corresponded to the carbonyl, C=O stretching vibration, and C=C stretching vibration exhibited by a peak at 1630 cm^−1^ that is characteristic of the unoxidized graphitic domain [29]. The C-O stretching vibration was confirmed by peaks at 1197 cm^−1^ and 1055 cm^−1^, which can be attributed to the epoxy and alkoxy groups, respectively. There was a peak at 578 cm^−1^ corresponding to the epoxide groups that are located at the edge of the GO sheet. The presence of oxygen functional groups was validated by these observations. Further structural characterization proceeded using Raman spectra in Figure 1 (b and c).

The graphite in Figure 1B displayed a strong sharp peak at 1557 cm^−1^ of the G band for first order scattering of the E_2g_ phonon of sp^2^ C-atoms [30], and a weak peak at 1339 cm^−1^ corresponding to the D band attributed to the disorder or defects in atomic arrangement [31]. A broad peak at 2679 cm^−1^ of the 2D band (G’ band) was observed, which is an indication of the number of layers present in the structure [32]. In comparison, GO (Figure 1C) had G and D bands that appeared to have right shifted. The spectrum was characterized by a G band at 1590 cm^−1^ and a D band at 1352 cm^−1^. Furthermore, there was an observed peak for the 2D band at 2436 cm^−1^. The 2D band present in GO may be an indication of a few layers in the structure. The level of disorder in the graphite and GO measured by intensity ratio (I_D_/I_G_) increased from 0.24 to 1.00. The observation is an indication of the decrease in size and crystallinity of graphitic material during chemical oxidation, thereby implying a high defect density of the amorphous carbon structure, whilst, at the same time, the increase in that of GO corresponded to a low-density regime [33].

**Figure 2:**
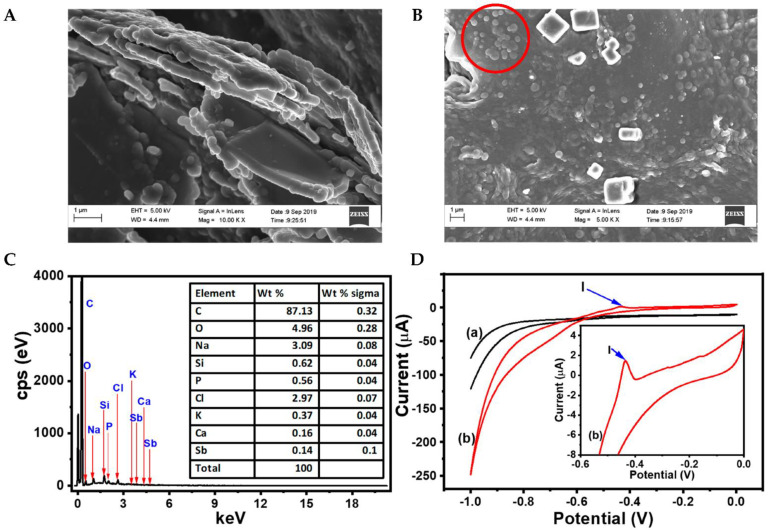
HRSEM of ERGO (**A**) and ERGO-SbNPs (**B**) on PGE surface, the accompanying EDX data in (**C**) showing elemental composition by weight%, and CV in (**D**) with comparison of ERGO-PGE (a) and ERGO-SbNPs-PGE (b) along with an inset of the magnified (b) in 0.5 mol·L^−1^ HCl solution at a scan rate of 50 mV·s^−1^.

The morphological analysis of graphite and the prepared GO proceeded using TEM and HRTEM. Graphite and GO prepared solutions were drop-casted onto Cu grids for HRTEM analysis. In Figure 1D, the graphite image displays dark flakes with no distinct shape and a straight edge. However, it can be observed that the GO layer (Figure 1E) was delaminated, and the separate sheet was apparent, which can be attributed to chemical exfoliation [29]. There was some wrinkling or folding of the observed transparent layer, which is due to low thickness and an indication of monolayer GO [34]. Selected area electron diffraction (SAED) (Figure 1F) was performed and the pattern had clear bright visible spots forming a hexagon, and thus confirming the hexagonal lattice structure of GO [29]. The electrochemical reduction of GO-Sb dispersion on a PGE to achieve ERGO-SbNPs-PGE was examined using an HRSEM. Figure 2 shows the HRSEM images of ERGO-PGE (a) and ERGO-SbNPs-PGE (b). The micrograph of ERGO-PGE (a) showed flake-like structures with a smooth surface attributed to reduced graphene oxide sheet stacking. In contrast, the HRSEM image of ERGO-SbNPs-PGE (Figure 2B) appears to have deposits of spherical protrusions displayed within the graphene sheets along with cubic structured deposits. The spherical structures indicated in the red circle are attributed to the formation of SbNPs with sizes ranging between 100 and 300 nm. It was further observed that unreacted metal salt deposits, shown by the cubic structured features, were still present and could be further removed or purified following further washing of the modified electrode surface prior to analysis. The accompanying energy dispersive X-ray analysis (EDX) of the formed ERGO-SbNPs-PGE with a table (insert) showing elemental composition (Figure 2C) was used to confirm the presence of Sb within the electrochemically formed complex. The EDX analysis confirms the inclusion of Sb within the complex in a low concentration of 0.14% wt. The low concentration of SbNP occurs due to low intercalation of the metal salt between the electrochemically reduced graphene oxide sheets during electrodeposition. This low yield of SbNP was, however, found to be optimum to reduce Sb-film production, and improve the electrode active surface area and electro-transfer kinetics of the modified electrode and sensitivity for PC detection.

Furthermore, the CV of ERGO-PGE and ERGO-SbNPs-PGE in 0.5 mol·L^−1^ HCl at a scan rate of 50 mV·s^−1^ is shown in Figure 2D. A single oxidation peak at a potential of −0.44 V (−440 mV), indicated by (I), was observed during the anodic scan of the latter. This corresponds to the oxidation of SbNPs from Sb^0^ to Sb^3+^, and a slight bump between −0.627 (−627 mV) and −0.778 V (−778 mV) on the reduction scan. A magnified image is shown in the inset provided. The electrochemical findings confirm the presence of SbNPs within the ERGO structure on the modified electrode surface. Similarly, Cesarino et al. [21] confirmed the presence of SbNPs on rGO by SEM and CV at −34 mV/−284 mV [21]. The observations in the current study indicate that the single-step method was able to produce ERGO-SbNPs by electrochemical reduction using CV under optimized CV parameters and number of deposition cycles. 

### 3.2. Electrochemical Properties

**Figure 3:**
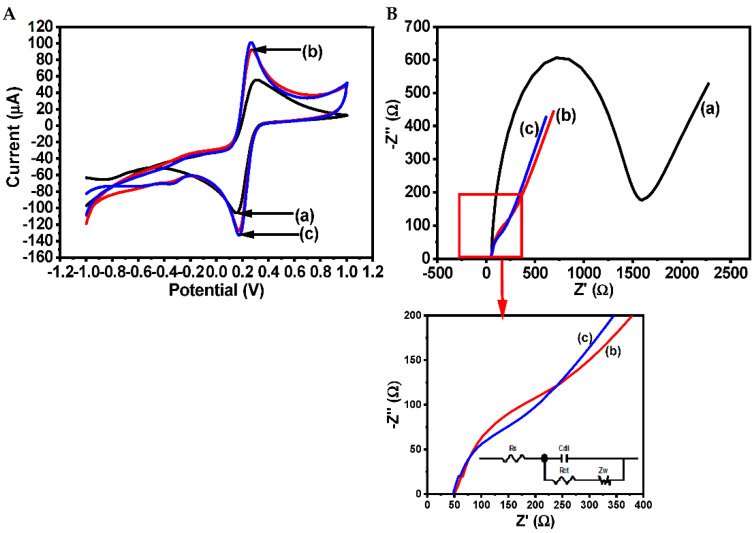
Electrochemical characterization of electrodes; (**A**) cyclic voltammograms of [Fe(CN)_6_]^3−/4−^ redox couple in 5 mmol·L^−1^ K_3_[Fe(CN)_6_] in 0.1 mol·L^−1^ KCl solution at bare PGE (a), ERGO-PGE (b), and ERGO-SbNPs-PGE (c) at a scan rate of 50 mV·s^−1^, and the corresponding Nyquist plot (**B**) from the peak currents.

The electrochemical characterizations of bare PGE, ERGO-PGE, and ERGO-SbNPs-PGE were carried out in 5 mmol·L^−1^ K_3_[Fe(CN)_6_] in 0.1 mol·L^−1^ KCl solution by CV with a scan rate of 50 mV·s^−1^ in the established potential range of −1.0 to +1.0 V vs. Ag/AgCl (3 mol·L^−1^ KCl). Figure 3A shows the results of the experiments. The redox peak currents of [Fe(CN)_6_]^3−/4−^ were improved with each modification from bare PGE (curve a) to ERGO-SbNPs-PGE (curve c) as shown in Figure 3A. The activity between the electrolyte and electrode surface is shown in Equation (1)

[Fe(CN)_6_]^4−^ ⇋ [Fe(CN)_6_]^3−^ + e^−^, (1)

where the peak currents’ ratio was determined as follows: bare PGE (curve a) had I_pc_/I_pa_ = 1.3, ERGO-PGE (curve b) had I_pc_/I_pa_ = 1.2, and ERGO-SbNPs-PGE (curve c) had I_pc_/I_pa_ = 1.08. The peak separation (ΔE_p_) values observed were greater than 0.059/n V, and they decreased in the following order: 0.155 V > 0.104 V > 0.090 V, respectively. From these observations, the I_pc_/I_pa_ ratio and ΔE_p_ values indicate an induced faster electron transfer of the [Fe(CN)_6_]^3−/4−^ redox couple on the electrode surface due to the electrocatalytic effect of ERGO and SbNPs, which improves the electrochemical signal on the electrode surface [19].

The corresponding EIS study proceeded with the investigated electrodes, and Nyquist plots are shown in Figure 3B. The bare PGE shown in (curve a) of Figure 3B presented a well-defined semicircle that corresponded to a higher R_ct_ value of 1368 Ω. Modification of the electrode with ERGO (curve b) showed a decreased R_ct_ and further so for ERGO-SbNPs-PGE (curve c) with values of 362.8 Ω and 236.9 Ω, respectively. The inclined line at a higher region of the Z’-axis was attributed to the Warburg impedance due to the diffusion of [Fe(CN)_6_]^3−/4−^ to and from the electrode surface, suggesting that the kinetics of the electrode process were diffusion-controlled at a higher region of the Z’-axis and controlled by charge transfer at a lower region of the Z’-axis [35]. Based on the observed values from the bare PGE to the ERGO-SbNPs-PGE, these indicate that charge transfer becomes faster with less resistance in modified electrodes.

### 3.3. Electrooxidation of PC on ERGO-SbNPs-PGE

**Figure 4:**
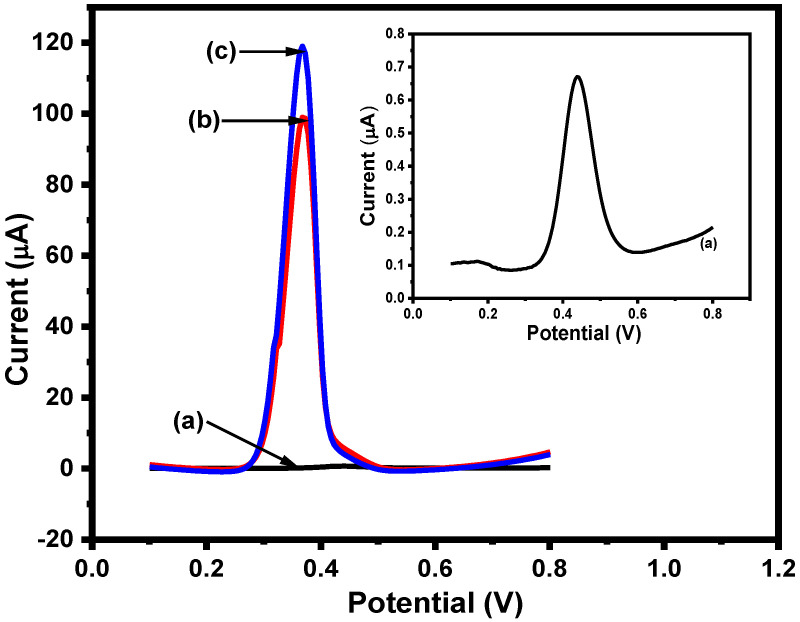
Differential pulse voltammograms of bare PGE (a), ERGO-PGE (b), and ERGO-SbNPs-PGE (c) in 0.1 mol·L^−1^ phosphate buffer solution at pH 7.0 containing 10 µmol·L^−1^ PC solution. Insert: DPV of bare PGE (a).

**Scheme 1:**
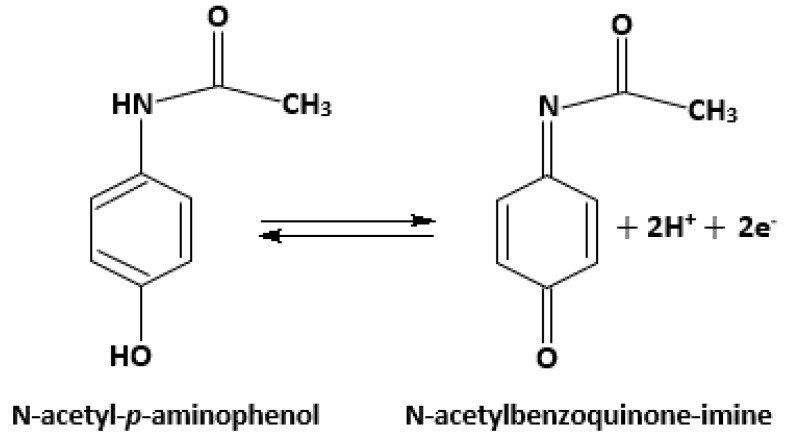
The electrochemical oxidation mechanism for PC (N-acetyl-*p*-aminophenol) Reprinted/adapted with permission from Ref. [9]. © 2022, IEEE.

Electrochemical oxidation of PC on the electrochemically characterized electrodes is shown in Figure 4. The DPV experiments were carried out in the potential range from 0.0 to +1.0 V vs. Ag/AgCl (3 mol·L^−1^ KCl) in a 0.1 mol·L^−1^ phosphate buffer solution at pH 7.0 containing a 10 µmol·L^−1^ PC solution. The experiments were conducted on three different electrodes, namely, bare PGE (curve a), ERGO-PGE (curve b), and ERGO-SbNPs-PGE (curve c). The oxidation peaks for PC on the electrodes were at the following potentials: bare PGE = 0.438 V, and ERGO-PGE and ERGO-SbNPs-PGE = 0.368 V, corresponding to the oxidation of PC as shown by the mechanism in Scheme 1. The shift in peak potential (from curve a to curve c) was noticeable with the modification of the PGE, which was an indication of the ease of electrooxidation for PC due to the increased signal response and electrocatalytic effect of ERGO and SbNPs. In Figure 4, the ERGO-PGE (curve b) showed an enhancement in the peak current from bare PGE (curve a). The conversion of GO to rGO improves conductivity by removing oxygenated species from the graphene and preventing possible unwanted reactions and electrostatic adsorption [22]. The greatest enhancement was shown by ERGO-SbNPs-PGE (curve c) by a factor of 1.2 in the peak current compared to that of ERGO-PGE (curve b). The enhancement is attributed to the synergistic incorporation of ERGO and SbNPs, which facilitated the electrooxidation of PC on the ERGO-SbNPs-PGE.

### 3.4. Effect of pH

**Figure 5:**
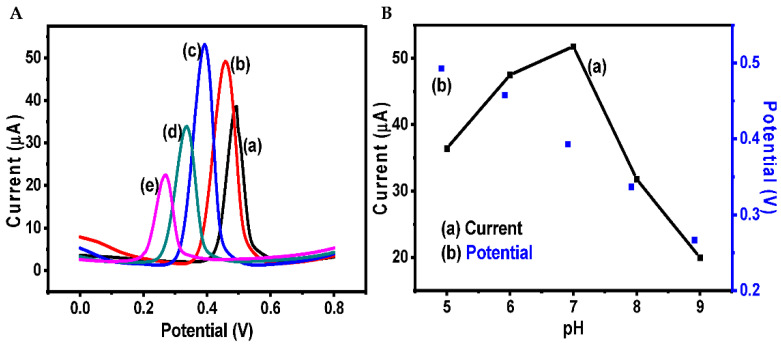
Effect of pH on the electrooxidation of PC on ERGO-SbNPs-PGE; DPV responses (**A**) of different pH values; 5.0 (a), 6.0 (b), 7.0 (c), 8.0 (d), and 9.0 (e). Linear plots of peak current (a) and potential (b) as a function of pH in (**B**) in 0.1 mol·L^-l^ phosphate buffer solution containing 10 µmol·L^−1^ PC in solution at various pH values.

The electrooxidation of PC was investigated at various pH values in a 0.1 mol·L^−1^ phosphate buffer solution containing 10-µmol·L^−1^-PC solutions. The pH values were varied from 5.0 to 9.0, which is where the oxidation product NAPQI shows stability, and DPV scans are shown in Figure 5A and the linear plots in (B). In Figure 5B, E_pa_ (curve a) shifted negatively with increasing pH. The linear relationship between the peak potentials and pH is given by the regression equation

E_pa_ (V) = −0.0578 pH + 0.7961 (R^2^ = 0.9861),

where the slope of −0.0578 V/pH that was obtained was found to be very close to the Nernstian slope of −0.059 V/pH for the same number of electrons and protons involved in the process [36]. This was observed in the oxidation mechanism of PC (Scheme 1) in which two electrons and two protons participated in the reaction. Peak currents changed with increasing pH, as shown in (curve a) of Figure 5B. For the investigated pH range of 5.0 to 9.0, the slightly acidic end (5 and 6) produced lower oxidation peak currents. This is due to the hydrolysis of PC in which the participation of protons (H^+^) with the unstable oxidation product NAPQI leads to p-aminophenol being formed [10]. In the more alkaline pH region (8 and 9), the involvement of hydroxide ions (OH^-^) in the reaction results in the instability of NAPQI, which in turn also yields lower peak currents [37]. The optimum pH at which PC underwent electrooxidation was pH = 7, which is where the electrochemical behaviour of PC is highly active [38], and this pH is very close to physiological conditions. Therefore, pH = 7 was used for the rest of the experiments involved in the study.

### 3.5. Reproducibility, Repeatability, and Stability

**Figure 6:**
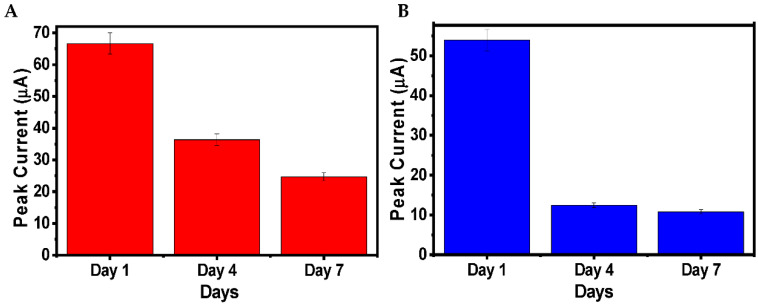
Bar graph of peak current measurements obtained on the same (**A**) and individual (**B**) ERGO-SbNPs-PGEs in 10 µmol·L^−1^ PC solution in 0.1 mol·L^−1^ phosphate buffer solution for repeatability, reproducibility, and stability over day 1, day 4, and day 7.

A series of experiments were performed to investigate the repeatability, reproducibility, and stability of ERGO-SbNPs-PGE as a sensing platform for PC detection. Measurements proceeded on the same and multiple electrodes, over a period of 7 days. The 7-day period included experiments that were performed on the same day (intra-day), and the rest on different days separated by two days in between (inter-day). All electrodes used for these experiments were prepared on the first day of measurements (day 1). Five experiments were performed on the same electrode and also on five individual electrodes in 10 µmol·L^−1^ PC in 0.1 mol·L^−1^ phosphate buffer solution (pH 7.03) over a potential range of 0.0 to 1.0 V vs. Ag/AgCl (3 mol·L^−1^ KCl) for DPV conditions. The results are shown in Figure 6 for the same ERGO-SbNPs-PGE (curve a) and individual ERGO-SbNPs-PGEs in (curve b) and are also accompanied by Table 1.

A relative standard deviation (RSD) of 0.803% for five measurements performed on the same ERGO-SbNPs-PGE on day 1 was obtained (Table 1). Day 4 and day 7 had RSDs of 0.98 and 0.95% for the 10 µmol·L^−1^ PC solution on the same ERGO-SbNPs-PGE, respectively. Experiments performed on the multiple ERGO-SbNPs-PGEs showed RSDs of 2.003%, 2.93%, and 4.19% for the 10 µmol·L^−1^ PC solution on days 1, 4, and 7, respectively. These showed the good repeatability and reproducibility of the ERGO-SbNPs-PGE for PC detection and were relatively comparable with data obtained for PC on other similar electrodes [34,35]. However, the bar graphs in Figure 6 show that the peak currents decreased from day 1 to day 4 (45.4%) and day 7 (62.97%) for the same ERGO-SbNPs-PGE experiments. Similarly, individual ERGO-SbNPs-PGE measurements showed peak currents decreased by 76.99% from day 1 to day 4 and 79.96% from day 1 to day 7. The findings indicate that the ERGO-SbNPs-PGEs suffer from poor stability as they do not retain the peak current response from the initial experiments over a longer period of time (between days). This may be due to the ERGO-SbNPs-PGE interacting with oxygen from the surrounding air, thereby decreasing the activity of the electrode [39]. Therefore, fresh ERGO-SbNPs-PGEs were prepared daily for all experiments conducted in the study. Further studies on electrode stability and storage conditions are required for future studies.

### 3.6. Interference Studies

**Figure 7:**
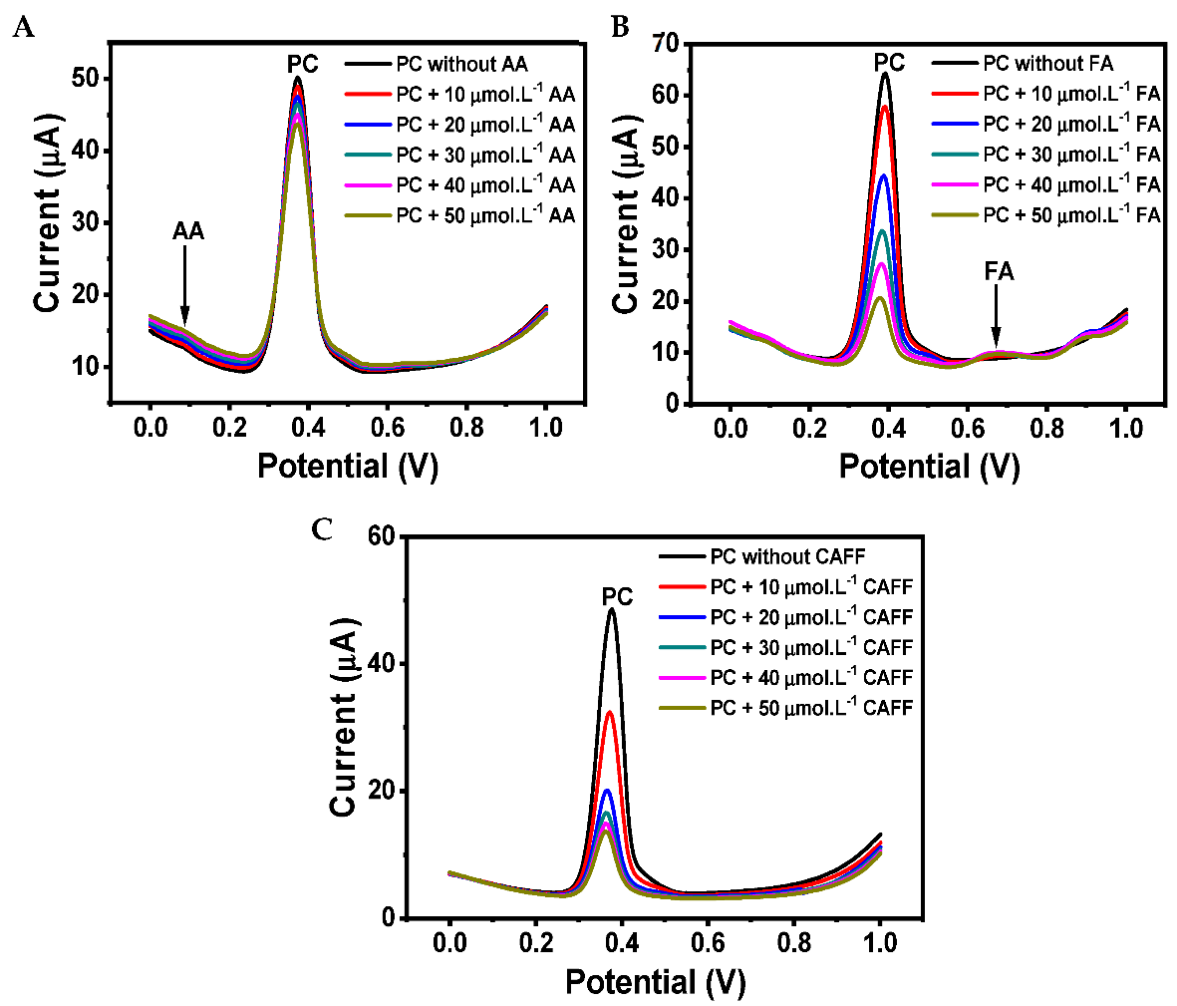
DPV results showing electrooxidation of 10 µmol·L^−1^ PC in 0.1 mol·L^−1^ phosphate buffer solution (pH = 7.03) at ERGO-SbNPs-PGE in the presence of interferents: (**A**) AA, FA (**B**), and CAFF (**C**) at different concentrations.

The selectivity of ERGO-SbNPs-PGEs towards PC was investigated in the presence of ascorbic acid (AA), folic acid (FA), and caffeine (CAFF). These have been reported to interact with paracetamol in pharmaceutical and commercial formulations [38,40,41]. Therefore, DPV measurements proceeded in a solution containing 10 µmol·L^−1^ PC with varying concentrations of interferent (10–50 µmol·L^−1^) in the potential range 0.0 to 1.0 V, and the results are shown in Figure 7 with the accompanying Table 2.

Figure 7 shows the peak potential for PC found at 0.383 V relative to those of the interferents: AA in (a) and FA (b), which were found at 0.0906 V and 0.665 V, respectively. Caffeine peak potential was reported outside the investigated potential range at 1.30 V. Ascorbic acid had an RSD of 6.08% (n = 3) as displayed in Table 2. This indicated interference, although slight, when compared to the findings in Table 1. The relative standard deviations (RSDs) for FA and CAFF were at 29.3% and 26.1%, respectively. Hence, these were highly interfering considering the effect of their presence on the peak current of PC. Such an occurrence may be due to electrode complexity and that FA and CAFF, or their respective oxidation products, may interact with the electrode surface.

### 3.7. Limit of Detection

**Figure 8:**
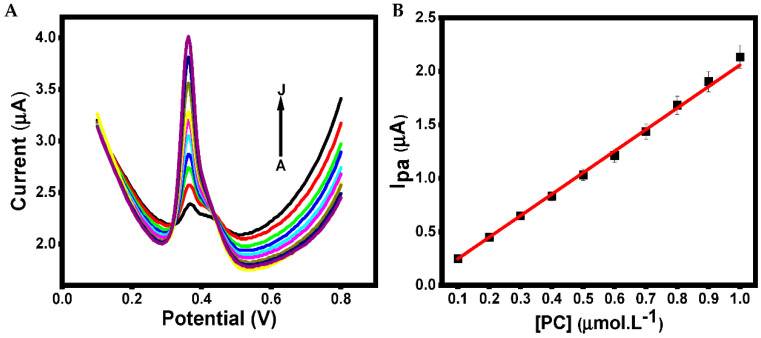
DPVs obtained at ERGO-SbNPs-PGE in 0.1 mol·L^−1^ phosphate buffer solution (pH 7.01) containing concentrations of PC (A–J: 0.1, 0.2, 0.3, 0.4, 0.5, 0.6, 0.7, 0.8, 0.9, and 1.0 µmol·L^−1^) in (**A**) and the corresponding calibration plots in (**B**).

The analytical performance of the ERGO-SbNPs-PGE was investigated to establish a linear concentration range and a limit of detection. The voltammograms for PC determination between 0.1 and 1.0 µmol·L^−1^ are shown in Figure 8A. An increase in the peak current for the oxidation of PC is seen with increasing PC concentration. Calibration studies were constructed from the average values of data collected from the three experiments conducted for each developed ERGO-SbNPs-PGE. Figure 8B shows a linear calibration plot of the peak current vs. the concentration of PC at different PC concentrations in the linear dynamic range in the region a 0.1–1.0 µmol·L^−1^ PC solution. The linear regression equation for the established region on an ERGO-SbNPs-PGE was as follows

I_pa_ (A) = 2.08 × 10^−6^ [PC] (µmol·L^−1^) + 1.56 × 10^−8^ (R^2^ = 0.998),

The limit of detection for PC on ERGO-SbNPs-PGE was determined using the slope from the linear plot in the following equation

(2) $$\mathrm{Detection}\;\mathrm{limit}=\frac{3\sigma }{m},$$

where *σ* is the standard deviation obtained from 10 blanks and *m* is the slope from the calibration linear plot. The average detection limit was determined as 0.057 µmol·L^−1^ for PC on ERGO-SbNPs-PGE. Similarly, a previous study reported that a modified glassy carbon electrode was developed based on the synergistic combination of rGO and SbNPs, which was used for the determination of estriol in environmental samples. The LOD found for this nanocomposite was 0.5 nmol·L^−1^ estriol in the linear range of 0.2 to 1.4 µmol·L^−1^ [21]. Table 3 shows the comparison of the detection limit found on ERGO-SbNPs with those of other modified electrodes. This shows that the ERGO-SbNPs-PGE was comparable with other carbon electrodes with similar modification for the determination of PC.

### 3.8. Analytical Application of ERGO-SbNPs-PGE for PC

**Figure 9:**
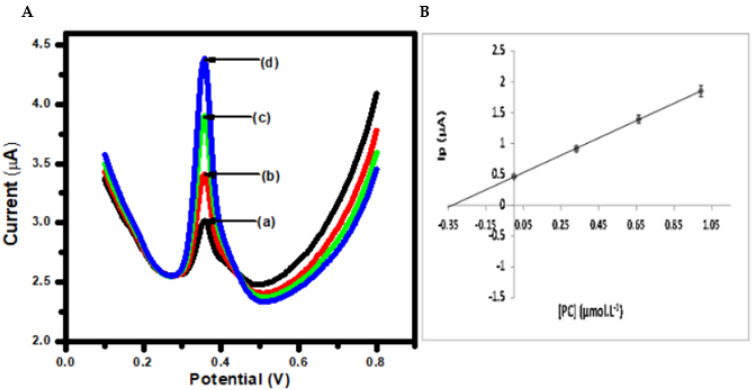
DPV measurements for the recovery of PC on ERGO-SbNPs-PGE with standard additions at different concentrations of sample (a); sample + 0.331 µmol·L^−1^ (b), sample + 0.662 µmol·L^−1^ (c), and sample + 0.993 µmol·L^−1^ (d) PC solution in (**A**), and linear plot of the peak current response of average standards added in (**B**).

The developed ERGO-SbNPs-PGE was applied in a real sample for analytical performance. Paracetamol samples were prepared by dissolving a tablet containing 500 mg PC in a 0.1 mol·L^−1^ phosphate buffer solution (pH = 7.01), and the recovery experiments were conducted using DPV. The resultant DPV measurements are shown in Figure 9A, and the corresponding linear plot in (b). Experiments were performed in triplicate and the sample was recovered by the use of standard addition. In the three determinations performed, Figure 9B shows a linear plot with average standard additions and the regression equation was obtained as

I_pa_ (A) = 1.403 × 10^−6^ [PC] (µmol·L^−1^) + 4.603 × 10^−7^ (R^2^ = 0.9998)

and the corresponding results in Table 4 show the recovery of an average ± standard deviation of 0.332 ± 0.0033 µmol·L^−1^ PC on the ERGO-SbNPs-PGE. For the added standards of 0.331, 0.662, and 0.993 µmol·L^−1^ PC, the recoveries were between 99.4 and 100.8% of PC in a commercial tablet. In addition, the calibration curve in Figure 8A shows the recovery of PC. A concentration 0.3 µmol·L^−1^ PC was used as it was similar to that of commercial 500 mg PC. The data in Table 4 show the recovery of an average ± standard deviation of 0.305 ± 0.03 µmol·L^−1^ PC with percentages between 99.7 and 101.6% on an ERGO-SbNPs-PGE. A significant difference was observed between the slopes of the calibration curve (2.08 × 10^−6^; R^2^ = 0.998) and the standard addition plot (1.403 × 10^−6^; R^2^ = 0.9998), as the former showed a significant error in which the matrix components may interfere with the peak signal in the determination of PC. Therefore, with this in consideration, the obtained recovery values of PC in a commercial tablet were accepted.

## 4. Conclusions

The incorporation of GO and the proposed SbNPs was implemented onto the PGE by electrodeposition to accomplish an ERGO-SbNPs-PGE sensor. The sensor was characterized electrochemically and thereafter used for the electrochemical determination of PC by use of adsorptive stripping differential pulse voltammetry. The electrochemical response was optimized to obtain the maximum enhancement of PC. The limit of detection was found to be 0.057 µmol·L^−1^, and this was comparable with previous similar electrodes. Standard addition and calibration curve methods were used in the quantification of PC in a pharmaceutical commercial tablet; the recovery was between 99.4 and 100.8% (n = 3).

## Figures and Tables

**Table 1:** The results below show the RSD% of peak currents obtained on specific days of experiments (1, 4, and 7) conducted on the same and individual ERGO-SbNPs-PGEs to investigate reproducibility, repeatability, and stability.

	Days	Relative Standard Deviation (RSD%) *
Same ERGO-SbNPs-PGE	1	0.803
	4	0.98
	7	0.95
Individual ERGO-SbNPs-PGEs	1	2.003
	4	2.93
	7	4.19

* RSD% of peak currents (n = 5).

**Table 2:** The results below show the effect of interferences, namely, AA, FA, and CAFF, on the oxidation peak current of PC at different concentrations and their RSD%.

Interferent	* C_i_ (µmol·L^−1^)	PC Peak Current (µA)	RSD%
AA	10–50	40.6 ± 2.47	6.08
FA	10–50	50.2 ± 14.7	29.3
CAFF	10–50	29.0 ± 7.56	26.1

* C_i_—concentration of interferent, peak current—average ± STDEV, RSD% of peak current (n = 3).

**Table 3:** Comparison of detection limits on ERGO-SbNPs-PGE with other modified electrodes at different pH and detection methods.

Modified Electrode	Detection Method	pH	Linear Range (µmol·L^−1^)	LOD (µmol·L^−1^)	References
Pd/GO/GCE	CV and DPV	6.8	0.005–0.5	0.002	[3]
RGO-Ag/CPE	CV and DPV	4.0	5.0–480.0	0.18	[10]
Fe_3_O_4_/rGO/Naf-GCE	DPV-ASV	6.0	2–10	0.17	[11]
Nafion/TiO_2_-Gr/GCE	CV and DPV	7.0	1–100	0.21	[17]
GCE/gCN-AgPVP	SQWV	6.1	0.2–100	0.079	[41]
Au-PEDOT/rGO/GCE	CV	7.0	0.001–8000	0.007	[42]
ERGO-SbNPs-PGE	AdsDPV	7.0	0.1–1.0	0.057	This work

**Table 4:** Recovery of PC in 500 mg commercial tablet using standard addition method and the use of calibration curve method from peak current response of concentrations.

Method	[PC]/µmol·L^−1^	Recovered [PC]/µmol·L^−1^	Recovery %
Standard Addition	0.331	0.329	99.4
	0.331	0.3336	100.8
	0.331	0.3338	100.8
Average ± STDEV	0.332 ± 0.003		
Calibration Curve	0.3	0.268	89.5
	0.3	0.304	101.3
	0.3	0.343	114.3
Average ± STDEV	0.305 ± 0.03		

STDEV—standard deviation, average ± STDEV (n = 3)

## Data Availability

Not applicable.
